# “Two scoops, please!”: Twin cryo-EM structures of an *Arabidopsis thaliana* DNA methyltransferase

**DOI:** 10.1093/plcell/koaf255

**Published:** 2025-10-23

**Authors:** Jan Wilhelm Hübbers

**Affiliations:** Assistant Features Editor, The Plant Cell, American Society of Plant Biologists; Unit of Plant Molecular Cell Biology, Institute for Biology I, RWTH Aachen University, Aachen 52056, Germany

If transcriptional enzymes such as RNA polymerases are vehicles on DNA roads, cytosine methylation is like placing cones on certain lanes: it slows or reroutes traffic rather than shutting the highway entirely. In this simplification, methyltransferase enzymes are the construction crews that place the methylation cones. Where and how this happens matters. DNA methylation marks promoters, long terminal repeats, transposable elements, and gene bodies, shaping expression and keeping transposable elements in check (Cokus et al. 2008).

The MET1 enzyme (UniProt P34881) maintains CG methylation in the model plant species Arabidopsis (*A. thaliana*). It belongs to the DNA methyltransferase (DNMT) family of enzymes that is largely conserved across eukaryotes (Schmitz et al. 2019). DNMTs have a characteristic domain architecture comprising a C-terminal methyltransferase (MTase) domain and regulatory N-terminal domains. For example, for human DNMT1 (*Hs*DNMT1; UniProt P26358), these include an replication-foci-targeting sequence (RFTS), a CXXC zinc-finger, and 2 Bromo Adjacent Homology (BAH) domains (Zhang et al. 2015; Fig. A). The MTase domain of *Hs*DNMT1 has an intrinsic substrate preference for hemi-methylated CG, while the RFTS domain autoinhibits DNMT1 in a DNA-competitive manner. This inhibition can be relieved via interactions involving histone H3. Unlike human DNMT1, *A. thaliana* MET1 has 2 N-terminal RFTS-like regions but lacks a canonical CXXC domain (Fig. B).

**Figure.**
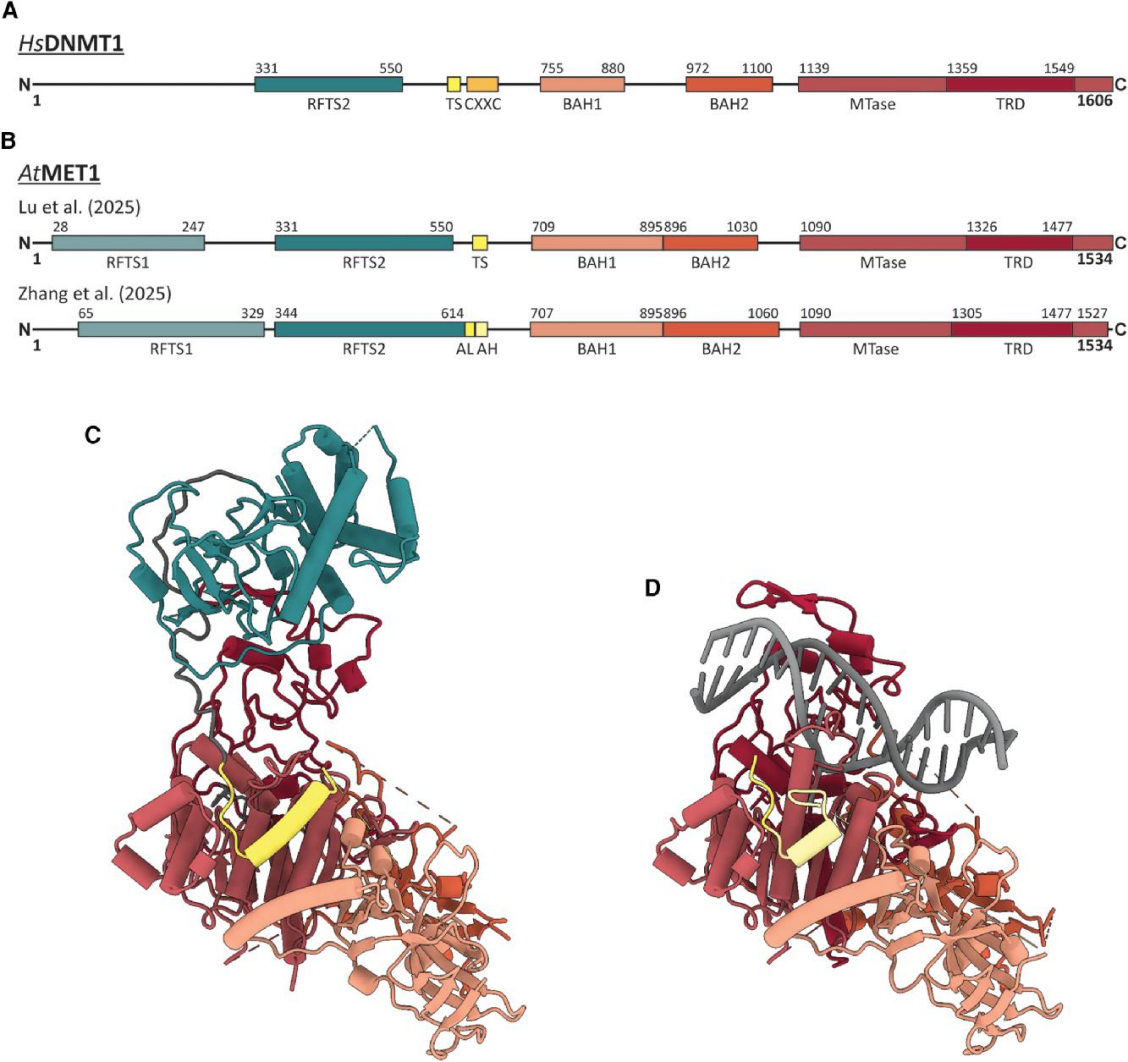
Domain architecture of human and *A. thaliana* methyltransferases and cryo-EM structures of *At*MET1. **A**, **B)** Domain architectures of human DNMT1 and *A. thaliana* MET1 as presented by Lu and co-workers (2025) and Zhang and co-workers (2025). Numbers indicate amino acids positions in the respective chains (not shown: DNMT1 TS (617–636), CXXC (646–692) and MET1 TS (625–646), AL (614–628), AH (629–646)). AH, activating helix; AL, autoinhibition loop; BAH, bromo-adjacent homology domain; CXXC, zinc finger domain; MTase, methyltransferase domain; RFTS, replication-foci-targeting sequence; TS, toggle switch. **C**, **D)** Cryo-EM structure of apo*At*MET1 as determined by Lu et al. 2025 (C) and *At*MET_ΔRFTS_-hmCG as reported by Zhang et al. 2025 (D). The.pdb files for structure visualization were kindly provided by the authors. The colors of the individual domains correspond to the color code in panel B.

In new work, **Jiuwei Lu and colleagues** (Lu et al. 2025) and **Zhihui Zhang and colleagues** (Zhang et al. 2025) report 2 cryogenic electron microscopy (cryo-EM) structures of *A. thaliana* MET1. The first structure, SAH-bound *At*MET1 (often termed “apoMET1” in the sense of lacking substrate), was solved at overall resolutions of 3.13 Å (Lu et al. 2025; Fig. C) and 2.92 Å (Zhang et al. 2025). In both apoMET1 structures, the N-terminal RFTS1 domain lacked density, probably due to its flexibility, and was not incorporated in the final model. The second structure, *At*MET_ΔRFTS_-hmCG, was deprived of its autoinhibitory RFTS2 domain to enable complexing with hemi-methylated CG and was solved at resolutions of 2.90 Å (Lu et al. 2025) and 2.34 Å (Zhang et al. 2025; Fig. D).

Both studies show that RFTS2 directly associates with the target-recognition subdomain (TRD) of the *At*MET1 MTase domain (Fig. B and C). The TRD helps grip DNA and position a flipped CpG cytosine so that the catalytic site can transfer a methyl group from SAM to the C5 position of the target cytosine in hmCG. By binding the TRD, RFTS2 blocks substrate recognition and autoinhibits MET1. The interface residues mediating this contact are conserved in plants, and targeted mutagenesis of several of them increases MET1 activity. Notably, the 2 groups altered different interface residues yet observed similarly increased *At*MET1 methylation activities (Lu: P361G or S401A/E1421A; Zhang: Y363A or N1430A/T1431A).

Structural alignment of *At*MET1 with *Hs*DNMT1 reveals high overall similarity between the 2 enzymes. However, *At*MET1 lacks *Hs*DNMT1's CXXC domain and its downstream inhibitory linker. In line with these architectural differences, the buried surface area at the RFTS–TRD interface is smaller in *At*MET1 than in *Hs*DNMT1. Lu and colleagues found that this difference affects the catalytic activity of both enzymes: whereas *At*MET1 had a higher catalytic activity at 4 °C, *Hs*DNMT1 was more active at 30 °C.

Both groups also report cryo-EM structures of *At*MET_ΔRFTS_ bound to its substrate hmCG DNA. Upon relief of RFTS2-mediated autoinhibition, the same regulatory helix—termed the toggle-switch (TS) by Lu and colleagues and the activating helix (AH) by Zhang and colleagues (Fig. B)—undergoes a pronounced conformational change that pushes the catalytic loop in the MTase domain toward the target cytosine and sets up the active site for methyl transfer.

Altogether, the similarity of these reports is a testament to the robustness of cryo-EM and current structural biology approaches. Reproducibility remains one of the highest quality hallmarks in science. In this light, it is also worth noting that a third report on the structure of *At*MET1 was recently published, further supporting the discoveries highlighted here (Kikuchi et al. 2025).

## Recent related articles in *The Plant Cell*

- Jiang et al. (2025) proposed a mechanism to ensure the development of precisely on megaspore mother cell in *A. thaliana* that relies on a balanced amount of methylated and demethylated DNA.
- Xin et al. (2024) reported cryo-EM structures of Alternative complex III that is essential for phototrophy in the bacterium *Chloroflexus aurantiacus*.
- Yao et al. (2024) showed that a 26S proteasome subunit degrades *At*MET1 to boost the expression of *TERMINAL FLOWER1*, maintaining indeterminate growth in the inflorescence meristem.
- Cao et al. (2023) presented DNA methylomes from nearly 100 accessions of rice to unravel the epigenetic basis of crop domestication and de-domestication.

## Data Availability

This article does not contain any primary data.

## Dive Curated Terms

The following phenotypic, genotypic, and functional terms are of significance to the work described in this paper:

MET1 Gramene: AT5G49160
MET1 Araport: AT5G49160

dna Gramene: EO:0007532
dna Araport: EO:0007532
